# Antimicrobial Stewardship Programmes in Saudi Hospitals: Evidence from a National Survey

**DOI:** 10.3390/antibiotics10020193

**Published:** 2021-02-17

**Authors:** Saleh Alghamdi, Ilhem Berrou, Zoe Aslanpour, Alaa Mutlaq, Abdul Haseeb, Mohammad Albanghali, Mohamed Anwar Hammad, Nada Shebl

**Affiliations:** 1 Department of Clinical Pharmacy, Faculty of Clinical Pharmacy, Albaha University, Albaha, Saudi Arabia; saleh.alghamdi@bu.edu.sa (S.A.); m.anwar@bu.edu.sa (M.A.H.); 2 School of Health and Wellbeing, Faculty of Health and Applied Sciences, University of the West of England, Bristol BS16 1DD, UK; 3 Department of Clinical, Pharmaceutical and Biological Sciences, School of Life and Medical Sciences, University of Hertfordshire, Hatfield AL10 9AB, UK; z.aslanpour@herts.ac.uk (Z.A.); n.a.shebl@herts.ac.uk (N.S.); 4 General Department of Pharmaceutical Care, Ministry of Health, Riyadh, Saudi Arabia; asmutlaq@moh.gov.sa; 5 Department of Clinical Pharmacy, College of Pharmacy, Umm Al Qura University, Makkah, Saudi Arabia; amhaseeb@uqu.edu.sa; 6 Department of Public Health, Faculty of Applied Medical Sciences, Albaha University, Albaha, Saudi Arabia; mohammad.aref@bu.edu.sa

**Keywords:** antimicrobial stewardship programmes, antimicrobial resistance, hospitals

## Abstract

Saudi hospitals and healthcare facilities are facing increasing rates of antimicrobial resistance and the emergence of new multi-drug resistant strains. This is placing an unprecedented threat to successful treatments and outcomes of patients accessing those facilities. The inappropriate use of antimicrobials is fueling this crisis, warranting urgent implementation of interventions to preserve antimicrobials and reduce resistance rates. Antimicrobial stewardship programmes (ASPs) can improve antimicrobial use, treatment success rates and reduce the levels of antimicrobial resistance. The Saudi Ministry of Health (MOH) devised a national antimicrobial stewardship plan to implement ASPs in hospitals, but little is known about the progress of implementation and the factors affecting it. This study aims to assess the level and the factors affecting the adoption and implementation of ASPs in Saudi hospitals at a national level. A nationwide cross-sectional survey was conducted in 2017 using an online questionnaire sent to all MOH hospitals. Overall, 147 out 247 MOH hospitals responded to the survey (54%). Only 26% of the hospitals reported the implementation of ASPs. Hospitals lack the knowledge, technological and staff resources to adopt and implement ASPs. Alternative models of ASP adoption could be explored to improve the rates of implementation of ASPs.

## 1. Introduction

Saudi Arabia is home to more than 10 million expatriates comprising more than 30% of the population, and an annual destination of more than 10 million people who travel from all parts of the world to Makkah and Medina for pilgrimage and Umrah [1]. The country is experiencing soaring rates of antimicrobial resistance (AMR) and emergence of rare and multidrug-resistant bacterial strains [2,3]. Because of the enormous potential of transmitting and globalising these novel multi-drug resistant strains, urgent action is needed to curb the rise of resistance rates and preserve the use of antimicrobials, which will soon cease to treat previously treatable infections [3].

The high prevalence of antimicrobial resistance is attributed to various factors, the most prominent of which are the misuse of antimicrobials [4,5], and the lack of antimicrobial stewardship programmes (ASPs) to ensure their judicious use [6,7,8]. ASPs are hospital-based programmes to improve antimicrobial use, optimise the treatment of infections and reduce adverse events associated with their use. These programmes can help increase infection cure rates, reduce treatment failures and increase the frequency of correct prescribing of antimicrobials for treatment and prophylaxis. They also significantly reduce hospital rates of *Clostridium difficile* infections and antibiotic resistance [9]. The Saudi Ministry of Health (MOH) devised a national antimicrobial stewardship plan in 2014 as part of the Arab Gulf regional strategy to reduce the threat of AMR, including the adoption and implementation of ASPs in MOH and private hospitals [10].

While there is evidence suggesting the implementation of ASPs in various Saudi tertiary hospitals and medical cities [6,7,11,12,13,14], there has never been a national study to assess the status of ASPs implementation in MOH hospitals, and to explore the factors that may affect it. This is important as implementation of national policies, such as the Saudi antimicrobial stewardship plan, often varies between hospitals of different type, depending on resources, reputation, local leadership, etc. In fact, little is known about the adoption of ASPs in smaller hospitals, and regions outside of the capital Riyadh, and the big cities. Furthermore, the published studies on the adoption of ASPs in Saudi hospitals do not include insights from hospital administrators, who would normally be involved in adoption decisions. Senior management support has often been reported as key to facilitating the adoption of ASPs in hospitals [15]. Exploring senior managers’ perspectives of implementing ASPs in hospitals would further clarify the factors that may facilitate or hinder implementation efforts. This study aims to explore the status of the adoption of ASPs in Saudi MOH hospitals, at a national level, and explore the factors that may affect their implementation. Knowledge of the status of and the factors that may affect ASP adoption at a national level may provide policymakers and commissioners with a better picture of the progress made and efforts needed to achieve the outcomes of the national antimicrobial stewardship plan. 

## 2. Results

### 2.1. Characteristics of the Responding Hospitals

Out of 274 Saudi MOH hospitals [16], 147 hospitals responded to the survey for an overall response rate of 53.6%. A total of 234 responses were received as on occasion, multiple administrators and healthcare professionals submitted their responses to the survey. Out of these responses, 20 were incomplete and were therefore excluded. We received 15 responses from CEOs and 8 responses from medical directors of the responding hospitals, only providing background information about their hospitals. These responses were considered as part of the demographics data. A total of 191 responses were included in the analysis of the factors influencing the adoption and implementation of ASPs in Saudi hospitals. Hospital pharmacists (33%) and pharmacy directors (26%) were the main respondents to the survey. Details of the respondents per profession are presented in Table 1.

Hospital pharmacists work in in-patient and out-patient pharmacies. They prepare and dispense medicines and carry out prescribing quality projects. Clinical pharmacists mainly work in the hospital wards carrying out daily ward rounds, reviewing and monitoring patients as part of the medical team.

Around 88% of responses were submitted by respondents from general hospitals (88%) from all regions across the Kingdom of Saudi Arabia. The types and number of the participants’ hospitals are presented in Table 2. The responses from each geographical region are shown in Table S1, which is included in Supplementary Materials.

### 2.2. Level of Implementation of ASPs in Saudi MOH Hospitals

The participants’ responses suggest that only 39 of the 147 MOH hospitals (26%) implemented ASPs; Of these hospitals, 33 are general hospitals, 5 obstetrics/gynaecology & paediatrics, and one rehabilitation hospital.

### 2.3. Factors Affecting the Adoption of ASPs in Saudi MOH Hospitals

We explored a number of factors that may influence ASPs adoption and implementation in Saudi MOH hospitals. These include: the perceived benefits of ASPs, the ease of their implementation, perceived demand pressures, the availability of resources, and the intention to adopt and implement ASPs.

#### 2.3.1. Perceived Benefits and Usefulness of the ASPs

As shown in Table 3, the participants are convinced of the benefits of ASPs in improving the use of antimicrobials, reducing antimicrobial resistance, and enhancing patient safety and care.

#### 2.3.2. Perceived Ease of Use

The participants are also convinced that adopting and implementing ASPs will be easy (57%), and that ASPs will be easy to follow and adhere to (61%). However, 82% of the responders from the participating hospitals do not know how to adopt and implement ASPs as shown in Table 4.

#### 2.3.3. Visibility of ASPs in Other Hospitals

Around 64% of the participants reported being aware of other hospitals in the country adopting ASPs. However, 51% remained neutral and 40% agreed (or strongly agreed) that ASPs were not very visible in other hospitals. This is shown in Table 5.

#### 2.3.4. Demand for and Pressure to Adopt ASPs in Hospitals

Hospitals adopt and implement interventions in response to patients’ demands, and external legislative pressures. As shown in Table 6, more than 50% of hospital responders consider the adoption of ASPs a patients’ expectation, which may place pressure on organisations to adopt this intervention. However, the majority of participants report not being pressured by the legislation to adopt and implement an ASP (73%) or to adhere to such legislation (83%).

#### 2.3.5. Readiness to Adopt and Implement ASPs

Around 85% of the respondents from the MOH hospitals reported that they do not have the knowledge and the technological resources needed to adopt and implement ASPs in their organisation. Furthermore, only 36% of the respondents thought their organisation has the financial resources needed as shown in Table 7. In relation to ASP teams/staff who would lead on the adoption and implementation of ASP, 82% of participants reported lack of necessary staff resources, and 77% reported lack of specific ASP staff/teams to champion the adoption and implementation of ASP in their organisation. This is not surprising given that only half the participants reported that their hospitals employed microbiologists (78/147), 33% employed antimicrobial pharmacists (48/147) and 31% employed ID specialists/consultants (46/147). Infection control practitioners were employed in 96% of the responding hospitals (141/147). 

Despite the reported lack of specialist staff, the majority of the respondents agreed that senior management is supportive of ASP adoption.

#### 2.3.6. Hospitals’ Intention to Adopt and Implement ASPs

Despite the low implementation of ASPs across MOH hospitals, the majority of participants confirmed their hospital’s intention to adopt and implement ASP as presented in Table 8. Around 87% of the participants reported that their hospitals would trial ASPs first before fully adopting them across the organisation.

Logistic regression was used as the dependent variable was dichotomous (nonadopters vs. adopters) to determine the effect of the various independent variables (ASP teams, perceived patients’ demand, senior management support, organisation readiness, external legislative pressure, trialability and usefulness of ASPs) on the hospitals’ intention to adopt them. Perceived patients demand, external legislative pressure and usefulness of ASPs were significantly related to hospitals’ intention to adopt ASPs. Organisation readiness, and trialability of ASPs were significant but negatively related to the hospitals’ intention to adopt ASPs. ASP team members and senior management support of ASP adoption did not exhibit a significant relation with hospitals’ intention to adopt ASPs. Therefore, we concluded that organisation readiness, trialability of ASPs, ASP team members and senior management support do not positively affect the hospitals’ intention to adopt ASPs. Their intention to adopt ASPs is rather driven by perceived patients’ demand and external legislative pressure. Tables S2 and S3 in Supplementary Materials show the results of the logistic regression analysis and the discriminating power of the model.

## 3. Discussion

This study explores the level and the factors affecting the adoption and implementation of ASPs in Saudi hospitals. To our knowledge, this is the first study exploring the levels of ASP adoption in Saudi hospitals at a national level. Antimicrobial stewardship programmes are implemented only in 26% of Saudi MOH hospitals. Even though senior management does not object to implementing ASPs, the hospitals lack the knowledge, technological and staff resources to adopt and implement ASPs. Despite the low levels of ASPs implementation, Saudi hospitals exhibit a strong intention to adopt them. Perceived patients’ demand and legislation strongly influence the hospitals’ intention to adopt and implement ASPs.

A recent survey of the adoption of ASPs in Nigerian tertiary hospitals reported similarly low levels of ASP adoption (24–35%) [17]. The levels of the adoption of ASPs in hospitals vary globally. the adoption of ASPs in US and European hospitals is amongst the highest in the world [18,19]. Elsewhere, despite the repetitive calls for better antimicrobial stewardship, the adoption of ASPs is still lagging behind, particularly in regions with a high burden of antimicrobial resistance, such as the Middle East [6,20]. Latin America [21], and Sub-Saharan Africa [22].

Saudi hospitals acknowledge the growing issue of antimicrobial resistance and the role of ASPs in tackling the issue. However, they do not know how to implement ASPs. This lack of knowledge is particularly exacerbated by the fact that adopting hospitals are not clearly setting example, since ASPs are not visible/ recognizable, and therefore, not generalisable and transferrable. Hospitals in Saudi Arabia which already found a way to implement ASPs could save other hospitals “the trouble of reinventing the wheel” through the dissemination of good practice [23]. Future research could evaluate the implementation process and outcomes, and how ASPs, as an organisation intervention, can be generalisable and transferrable within the Saudi context.

Adopting and implementing ASPs in hospitals is a complex endeavor, requiring financial and human resources [23] as well as technology resources [24]. Confirming our previously reported barriers [7], the surveyed hospitals reported lack of necessary knowledge, technology and human resources to adopt ASPs. These barriers have also been reported in other contexts; Kapadia et al. reported that the lack of integration of IT resources into daily workflow can hinder ASP implementation [25]. However, in Nova Scotia hospitals, the efficient use of information technology can improve antimicrobial stewardship practices [26]. Lack of funding remains a commonly reported obstacle to ASPs adoption; Beovic et al. international survey found that only a minority of countries had dedicated ASPs funding [27]. However, the major barrier to ASP adoption across continents and healthcare settings is the lack of ASPs teams and ID specialists [18]. Alternative ASP implementation models have been suggested to address this challenge [28,29,30]. Senior management in Saudi hospitals is supportive of the adoption of ASPs and implementation; this is a key facilitator, as reported in Maki et al.’s feasibility study [31], to ensure resource allocation, and ASP monitoring and evaluation. Senior managers’ support needs to extend beyond the “no objection to implementation” stance, to actively secure the necessary funds, skills and expertise to implement ASPs. Future research could qualitatively explore how the adoption of ASPs barriers have been addressed in hospitals with similar context.

The intention of Saudi hospitals to adopt and implement ASPs is not affected by the (lack of) ASP teams, senior management support, organisation readiness, trialability and usefulness, as these were reported to affect the actual adoption and implementation process. The intention of Saudi hospitals is strongly influenced by the strict enforcement of legislation and perceived patients demands/expectation. We previously reported that the lack of enforcement of policies and guidelines from the MoH and hospital administration remains a significant barrier to ASP adoption and implementation [7]. This barrier has also been highlighted in Maki et al.’s 2020 study [31]. MOH hospitals are clearly expecting the MOH to enforce the adoption, implementation and regular monitoring of ASPs in its hospitals. Legislation mandating ASPs implementation is key to improving implementation rates, and subsequently, appropriate antimicrobials’ use [32]. The MOH could set up a hospital accreditation system, to mandate hospitals to implement ASPs and report on their key performance indicators. Saudi hospitals are already familiar with accreditation requirements as a few seek the Joint Commission International accreditation, which mandates ASP implementation. Future research could explore policymakers and hospital CEOs perspectives on implementing ASPs in hospitals and how organisations like the MOH could collaborate with stakeholders to improve the rates of ASPs adoption in hospitals and other healthcare settings.

Patient demand as a significant motivator for the adoption of ASPs in hospitals is a complex factor [33]—in our study, this relates to the hospital’s image/reputation. Top-performing/advanced hospitals tend to have ASPs in place [34]. In Saudi hospitals, the hospital respondents perceive ASPs as part of patient care [35]. They also consider adopting and implementing ASPs an advanced practice that will convince patients they are being treated in a top-ranking hospital, and receiving high-quality service; this, in turn increases the patients’ satisfaction and trust in the hospital and the clinicians working there. Saudi hospitals adopting ASPs provide better patients’ antimicrobial stewardship education than non-adopters [36]. However, patients (globally) remain marginally involved in hospitals’ adoption and implementation of ASPs [34] despite being “the receiver” of ASPs outcomes. Future research could explore how patients could be engaged in the adoption of ASPs and implementation process, and how this engagement could be translated into measurable programme outcomes.

### 3.1. Research Implications

The findings of this study provide practical implications to healthcare professionals, hospital administrators and policymakers. First, legislation mandating the adoption of ASPs and adherence to ASPs policies and guidance is crucial to improving antimicrobials’ use in hospitals and reducing the burden of antimicrobial resistance. The MOH needs to actively support and oversee hospitals’ implementation of ASPs, and the reporting and monitoring of ASP outcomes as key performance indicators of care quality. This will enable hospitals and the MOH to assess if ASPs are delivering their intended benefits, and devise plans to address any shortcomings. Second, ASP implementation tools need to be developed with input from MOH, lead infectious disease specialists, hospital pharmacy and microbiology departments. Hospitals with established ASPs should share implementation processes and outcomes with hospitals in the region and nationally. Alternative ASP models should be explored to optimize the use of limited infectious disease expertise and microbiology facilities, perhaps through setting regional antimicrobial stewardship hubs where resources are accessible and shared across multiple hospitals. Third, Saudi hospitals should harness the advantages of information technology to improve antimicrobial prescribing practices, monitoring of antimicrobials’ use and tracking of outcomes of ASPs. This will enable benchmarking of performance against regional and national performance, and highlight areas requiring improvement.

### 3.2. Research Limitations

Several potential limitations must be considered when interpreting the findings of this study. First, this study only assesses the levels of ASP adoption in hospitals, and does not report on the outcomes of these programmes. Future research could explore ASP reported outcomes such as impact on antimicrobial usage, and rates of antimicrobial resistance. Second, we received a low response from CEOs and medical directors, and their responses only covered the status of ASP implementation, availability of ASP team members and the hospital’s intention to implement ASPs. Since they represent an important link between policy and practice, future research could explore their input on further factors that could influence ASPs implementation. Third, this study focused on MOH hospitals. It will be interesting to compare our findings with private hospitals’ adoption of ASPs. Fourth, the study collected cross-sectional descriptive data to illustrate the current status of the adoption of ASPs in hospitals and the factors perceived to hinder greater adoption. Future studies could collect longitudinal data to determine causal links between factors and outcomes more explicitly. Although our sample size was adequate, the findings might vary with larger samples.

## 4. Methods

### 4.1. Subjects and Setting

All MOH hospitals across the Kingdom of Saudi Arabia (274) [16] were included in this study. The hospitals were stratified, based on hospital type, into: general, psychiatric, obstetrics gynaecology and paediatrics, convalescence, eye, obstetrics, paediatrics, chest and rehabilitation hospitals.

### 4.2. Questionnaire Development, Validation and Piloting

A pilot survey was devised based on findings of prior research [7] and a thorough review of the literature on antimicrobial stewardship programmes in hospitals. The survey was reviewed for face and content validity by two infectious diseases consultants and two specialist antimicrobial stewardship pharmacists in Saudi Arabia, and one specialist antimicrobial stewardship pharmacist in the UK. Discriminant validity was conducted to confirm that the instrument can distinguish between the negative and positive views of the respondents [37].

The pilot questionnaire was sent to 60 participants (37 pharmacists, 12 infection control nurse specialists, seven microbiologists, and 4 infectious diseases consultants) from 5 MOH hospitals (1 ASPs adopting and 4 non-adopting hospitals), which were randomly selected. Minor modifications were made to the survey post-piloting. The final survey contained two main sections: background information about the hospital (14 items), and the factors that may affect the adoption and implementation of ASPs in the hospital (28 items). Closed questions and Likert-type statements were included to explore: intention to adopt ASPs (3 items), legislation and regulation relating to the adoption and implementation of ASPs (4 items), hospital characteristics (9 items) and ASPs characteristics (12 items). For Section 2, questions 15–30 were developed based on findings from prior research [7], and articulated using the determinants of innovation within the healthcare organisations model [38,39]. Questions 31–42 were developed and articulated based on the determinants of innovation within the healthcare organisations model [38,39].

Invitation letters explaining the study and including the online survey link were sent through internal MOH emails to the potential participants (CEOs, clinical and hospital pharmacists, infection control practitioners, ID physicians, medical directors, pharmacy directors and microbiologists) through the general directorate for research and studies at the Saudi MOH (targeting hospital CEOs and medical directors), the general department of pharmaceutical care at the Saudi MOH (targeting clinical and hospitals pharmacists and pharmacy directors) and the general department of health facilities infection control at the Saudi MOH (targeting infection control practitioners, ID physicians and microbiologists). The study was also advertised through social media platforms (WhatsApp and Twitter) using official WhatsApp numbers and official WhatsApp accounts of health region offices and hospitals. Phone calls were made at two weekly intervals to non-responding hospitals to encourage participation. CEOs and medical directors (with busy schedules) were only required to respond to the background section of the survey to maximise participation from this group. Data collection took place during May–August 2017.

### 4.3. Data Analysis

Data were extracted from the SurveyMonkey^®^ online tool, uploaded to the SPSS database (Version 23; SPSS Inc., NY, USA) and analysed using descriptive statistics (frequencies, percentages, mean and standard deviation). Logistic regression analysis was used, as the dependent variable was dichotomous (non-adopters vs. adopters), to determine the effect of the various independent variables on the hospital’s intention to adopt ASPs. *p* values *p* < 0.05 were considered statistically significant.

## 5. Conclusions

The adoption of antimicrobial stewardship programmes in Saudi MOH hospitals remains low. Lack of knowledge, infectious disease expertise, ASP teams, and technological resources have been suggested as reasons for the lagging adoption. Legislation mandating the adoption and monitoring of ASPs and patient expectations are strong drivers for their adoption. Understanding the key drivers for ASP adoption and why Saudi hospitals are lagging is crucial in the effort to improve the use of antimicrobials in hospitals and reduce antimicrobial resistance.

## Figures and Tables

**Table 1:** Survey respondents per profession.

Profession	Number of Reponses
CEOs	15 (7%)
Clinical pharmacist	20 (9%)
Hospital pharmacist	70 (33%)
Infection control nurse	12 (5%)
Infection control specialist/consultant	8 (4%)
ID specialist/consultant	17 (8%)
Medical director	8 (4%)
Microbiologist	8 (4%)
Pharmacy director	56 (26%)
Total	214 (100%)

**Table 2:** Survey responses per hospital type.

Hospital Type	Number of MOH Hospitals	Number of Participating Hospitals
General	218 (79.5%)	129 (88%)
Psychiatric	18 (6.5%)	5 (3%)
Obstetrics/Gynaecology & paediatrics	16 (6%)	9 (6%)
Convalescence	7 (2.5%)	1 (0.7%)
Eye	4 (1.4%)	1 (0.7%)
Other hospitals	9 (3%)	0
Rehabilitation	2 (0.7%)	2 (1.5%)
Total	274 (100%)	147

Other hospitals include (obstetrics/gynaecology, paediatrics, and chest).

**Table 3:** The participants’ perceived benefits and usefulness of ASPs.

Statements	Relative Frequency Distribution					Mean (sd)
	Strongly Agree %	Agree %	Neutral%	Disagree %	Strongly Disagree %	
ASPs will improve antimicrobial use	69.6	26.7	3.7	0.00	0.00	4.66 (0.547)
ASPs will reduce antimicrobial resistance	70.2	26.2	3.1	0.5	0.00	4.66 (0.566)
ASPs will improve patient safety	73.3	24.1	2.1	0.5	0.00	4.70 (0.533)
ASPs will not improve patient care	1.0	0.5	5.8	34.0	58.6	4.49 (0.724)

**Table 4:** The hospital participants’ perceived ease of use of ASPs.

Statements	Relative Frequency Distribution					Mean (sd)
	Strongly Agree %	Agree %	Neutral %	Disagree %	Strongly Disagree %	
I believe that it will be easy to adopt and implement ASP in this hospital.	13.1	43.5	22.0	16.8	4.7	3.43 (1.064)
Overall, I believe that ASP will be easy to adhere to	12.0	49.2	20.9	16.2	1.6	3.54 (0.955)
I believe that it will be easy for staff to follow ASP guidelines.	12.6	48.2	18.3	18.3	2.6	3.50 (1.015)
Procedures for adopting and implementing ASP are clear and understandable.	0.5	1.6	15.7	66.0	16.2	2.04 (0.656)

**Table 5:** The perceived adoption of ASPs in other hospitals.

Statements	Relative Frequency Distribution					Mean (sd)
	Strongly Agree %	Agree %	Neutral %	Disagree %	Strongly Disagree %	
ASPs are adopted in other hospitals in the country	14.1	49.7	31.9	3.7	0.5	3.73 (0.766)
ASPs are not very visible in other hospitals	6.8	33.0	51.3	8.9	0.00	2.62 (0.743)

**Table 6:** Perceived patients’ demands and legislative pressures to adopt ASPs.

Statements	Relative Frequency Distribution					Mean (sd)
	Strongly agree %	Agree %	Neutral %	Disagree %	Strongly Disagree %	
Many of our patients would expect this hospital to adopt and implement ASP	10.5	46.6	24.6	12.0	6.3	3.43 (1.038)
Our patients would consider us to be forward-thinking by adopting and implementing ASP	20.4	44.0	23.0	7.9	4.7	3.68 (1.036)
The legislative regulation pledges this hospital to adopt and implement ASP	0.5	7.9	18.3	52.9	20.4	2.15 (0.854)
The compliance with the legislative regulations regarding ASP is enforced strictly	0.0	2.6	14.1	61.8	21.5	1.98 (0.68)

**Table 7:** MOH hospitals and their readiness to adopt ASPs.

Statements	Relative Frequency Distribution					Mean (sd)
	Strongly Agree %	Agree %	Neutral %	Disagree %	Strongly Disagree %	
We have the knowledge necessary to adopt and implement ASP in this hospital	0.5	2.1	12.6	61.3	23.6	1.95 (0.70)
This hospital has the financial resources to adopt and implement ASP	8.4	27.7	30.9	22.0	11.0	3.01 (0.13)
This hospital has the technological resources to adopt and implement ASP	0.0	2.1	13.1	62.8	22.0	1.95 (0.66)
We have the resources necessary (e.g., experts and staff) to adopt and implement ASP in this hospital.	0.5	3.1	14.1	54.5	27.7	1.94 (0.77)
A specific person (or group) is available for assistance with the adoption and implementation of ASP in this hospital	1.6	5.8	15.7	53.9	23.0	2.09 (0.87)
Senior management would provide resources necessary for the adoption and implementation of ASP in this hospital	16.8	41.9	26.2	11.5	3.7	3.57 (1.02)
Senior management would provide necessary support for the adoption and implementation of ASP in this hospital.	15.7	45.0	24.6	10.5	4.2	3.58 (1.01)
Senior management would support adherence to ASP in this hospital.	15.7	51.3	23.6	6.3	3.1	3.70 (0.92)
Senior managers would be enthusiastic about adopting and implementing ASP in this hospital.	18.8	45.5	22.0	11.0	2.6	3.67 (099)

**Table 8:** Hospitals’ intention to adopt and implement ASPs (*n* = 191).

Statements	Relative Frequency Distribution					Mean (sd)
	Strongly Agree %	Agree %	Neutral %	Disagree %	Strongly Disagree %	
This hospital intends to adopt and implement ASP	45.5	36.1	14.7	2.6	1.0	4.23 (0.869)
This hospital intends to follow ASP guidelines regularly in the future	41.9	38.2	15.2	3.1	1.6	4.16 (0.904)
This hospital would highly recommend the adoption and implementation of ASPs in other hospitals	31.9	40.8	17.8	7.9	1.6	3.94 (0.977)
Before deciding whether to adopt ASPs, it will be essential to be able to properly try them out	28.3	59.2	8.9	3.1	0.5	4.12 (0.73)
It is essential to adopt ASP on a trial basis long enough to see their benefits	39.3	47.6	7.3	4.2	1.6	4.19 (0.86)

## Data Availability

Data available on request due to ethical restrictions. The anonymised data presented in this study are available on request from the corresponding author. The data are not publicly available to maintain privacy and adhere to guidelines of the ethics protocol.

## Supplementary Materials

The following are available online at https://www.mdpi.com/2079-6382/10/2/193/s1, Table S1: Survey responses per geographical region, Table S2: Results of the logistic regression, and Table S3: Discriminating power.
