# Supplementary material for: Antimicrobial Stewardship Programmes in Saudi Hospitals: Evidence from a National Survey
Source: Antibiotics (Basel). 2021 Feb 17;10(2):193. doi: 10.3390/antibiotics10020193 (PMC7923167; doi:10.3390/antibiotics10020193)
Supplement: Supplementary file 1 [file antibiotics-10-00193-s001.pdf]

antibiotics

MDPI

Supplementary Materials File

Table S1. Survey responses per geographical region.

Region	Number of MOH Hospitals	Number of Participating Hospitals
Al-Hasa	11	2 (1.3)
Al-Jouf	9	4 (3%)
Al-Qunfudah	5	1 (0.7%)
Al-Qurayyat	5	2 (1.3%)
Aseer	20	13 (9%)
Baha	10	5 (3.5%)
Bishah	7	4 (3%)
Eastern region	19	12 (8%)
Hafar Al-batin	7	5 (3.5%)
Hail	12	3 (2%)
Jeddah	13	10 (7%)
Jizan	21	6 (4%)
Madinah	19	15 (10%)
Makkah	9	5 (3.5%)
Najran	12	9 (6%)
Northern border	9	7 (5%)
Qasim	20	12 (8%)
Riyadh	42	21 (14%)
Tabouk	11	8 (5%)
Taif	13	3 (2%)
Total	274	147

Table S2. Results of the logistic regression.

Predictor	B Coefficient	Wald Statistic	Significance
TM	1.737	6.996	0.515
PD	0.867**	4.068	**0.002**
SMS	0.973	7.065	0.079
OR	−1.790*	5.935	**0.050**
EP	3.751*	10.303	**0.029**
TRI	−2.180**	8.016	**0.013**
UN	1.127**	6.424	**0.002**

Goodness-of-fit omnibus $\chi2 = 77.987$, df = 8, $p < .001$. TM = Team Members (item 26); PD = (perceived) Patients Demand (items 18 and 21); SMS = Senior Management Support (items 22–25); OR = Organisation Readiness (items 27–30); EP = External Pressure (items 19 and 20); TRI= Trialability (items 37 and 38); UN = Usefulness (39–42) * $p < 0.05$ ** $p < 0.01$.

Table S3. Discriminating power.

		Predicted		% Correct
		Non-adopters	Adopters	
Observed	Non-adopters	16	1	80.0
	Adopters	5	99	95.2
Overall				92.7

The research model has an overall predictive accuracy of 92%. Overall, the research model exhibits an acceptable fit with the data.
